# The Impact of COVID-19 on Orthopedic Surgery Fellowship Training: A Survey of Fellowship Program Directors

**DOI:** 10.1177/15563316211012006

**Published:** 2021-05-10

**Authors:** Braiden M. Heaps, Jeffrey R. Dugas, Orr Limpisvasti

**Affiliations:** 1 Kerlan-Jobe Orthopaedic Clinic, Los Angeles, CA, USA; 2 Andrews Sports Medicine & Orthopaedic Center, Birmingham, AL, USA

**Keywords:** COVID-19, orthopedic training, fellowship, education, program directors

## Abstract

*Background*: The COVID-19 pandemic has had a wide-reaching impact. Graduate medical education of orthopedic surgeons was not spared from the jarring changes. *Purpose*: We sought to survey fellowship program directors in the field of orthopedic surgery about how the COVID-19 pandemic affected the education of the 2019 to 2020 and 2020 to 2021 fellowship classes and the future of their programs. *Methods*: In October 2020, an 18-item survey was distributed by an official of the American Academy of Orthopedic Surgeons (AAOS) to the specialty societies that govern fellowship training. Each specialty society then distributed the survey to its respective program directors. A reminder email was sent during the enrollment period. Each respondent was able to complete the survey once. Survey questions were grouped into 3 sections: general information about the fellowship training programs, the impact of COVID-19 on the 2019 to 2020 fellowship class, and the future impact of COVID-19 on the fellowship training programs. *Results*: Of the 564 accredited orthopedic surgery fellowship programs in the United States, 190 directors responded. Of these, 73.59% reported COVID-19 had a negative impact on the 2019 to 2020 fellowship class. A normal distribution of responses was found regarding didactic and academic learning, research, and mentorship opportunities. A majority of respondents said they believe that there will be no negative impact on patient care the fellows provide in the years to come. *Conclusion*: Orthopedic surgery fellowship program directors acknowledged that while there were negative effects to training in the pandemic, they did not think these would negatively affect patient care provided by 2019 to 2020 fellows in the short and medium term. They also reported positive outcomes from the experience of the pandemic, including new ways to educate fellows.

## Introduction

The impact of the COVID-19 pandemic has been dramatic and far reaching, and graduate medical education of orthopedic surgeons has not been spared. Medical students looking to apply to competitive orthopedic residency programs have had to search for alternative ways to optimize their experience through, for example, developing mentorships with local orthopedic surgeons or physicians outside of orthopedics due to traditional methods of externships being discouraged [1,11]. Furthermore, multiple orthopedic surgery residency programs saw their trainees “repurposed” into emergency or critical care roles as the need emerged [6,8]. This occurred in the United States, as well as throughout Europe, South Korea, and India [3,4,7,12,14].

Studies have been published from the perspective of trainees in various phases of their medical education including students [11], residents [2], and fellows [9,13], but fewer studies have been published from the educators’ point of view [5,10,15]. We therefore sought to assess the effects of the COVID-19 pandemic on orthopedic surgery fellowship programs by surveying program directors of the 2019 to 2020 and 2020 to 2021 fellowship classes about the short- and long-term effects of COVID-19 on orthopedic fellows’ education. To our knowledge, this was the first study to survey orthopedic surgery educators exclusively about their experiences in the COVID-19 pandemic.

## Methods

We created an 18-item survey using SurveyMonkey (Palo Alto, California), a secure web application used for building and managing online surveys and databases. The study was approved by the institutional review board of our institution. On October 6, 2020, an official of the American Academy of Orthopedic Surgeons (AAOS) distributed the survey (Supplemental Table 1) to the specialty societies that govern orthopedic surgery fellowship training: American Association of Hip and Knee Surgeons (AAHKS), Arthroscopy Association of North America (AANA), American Orthopedic Foot and Ankle Society (AOFAS), American Orthopedic Society for Sports Medicine (AOSSM), American Shoulder and Elbow Surgeons (ASES), American Society for Surgery of the Hand (ASSH), Musculoskeletal Tumor Society (MSTS), North American Spine Society (NASS), Orthopedic Trauma Association (OTA), and the Pediatric Orthopedic Society of North America (POSNA). Each specialty society then distributed the survey to its respective fellowship training program directors. A reminder email was sent during the enrollment period, on October 26, 2020. The survey closed on November 6, 2020. Each respondent was able to complete the survey only once.

The survey questions were grouped into 3 sections: general information about the program, the effect of the COVID-19 pandemic on the education of the 2019 to 2020 fellowship class, and the possible effects of the COVID-19 pandemic on programs in the future. The first section consisted of multiple-choice questions, and the second and third sections asked participants to respond to questions and to statements, respectively, on a 5-point Likert scale.

At the time of the survey, there were 564 accredited orthopedic surgery fellowship training programs in the United States, which are governed by subspecialty societies. Each society oversees the following number of programs: AAHKS, 103; AANA/AOSSM, 88; AOFAS, 50; ASES, 31; ASSH, 90; MSTS, 20; NASS, 74; OTA, 62; and POSNA, 46. A survey was sent to the program directors of these fellowship programs.

## Results

Of 564 surveys sent, there were 190 respondents, for a 33.70% response rate. When broken down by subspecialty, directors of foot and ankle (62.00%) and pediatrics (60.87%) fellowship programs had the highest response rate, while hand and wrist (4.44%) had the lowest (Supplemental Table 2). Of the 190 programs, 145 (76.3%) trained only 1 or 2 fellows per year. The geographic composition of the responses was diverse, with every region in the country represented. Large academic centers, which hosted 62.4% of programs, represented the largest proportion of training programs (Supplemental Table 3).

When we asked about the impact of COVID-19 on the clinical and surgical training of 2019 to 2020 fellows, we received 178 responses. Of these, 3 (1.69%) responded “very positive,” another 3 (1.69%) said “somewhat positive,” 41 (23.03%) said “neutral,” 103 (57.9%) said “somewhat negative,” and 28 (15.7%) answered “very negative” (Supplemental Fig. 1).

When we asked about the impact of COVID-19 on the didactic and academic learning (non-clinical) of fellows in the 2019 to 2020 academic year, we received 178 responses. Of these, 14 (7.9%) responded “very positive,” 49 (27.6%) reported it was “somewhat positive,” 57 (32.0%) said it was “neutral,” 53 (29.8%) reported “somewhat negative,” and 5 (2.81%) thought the impact was “very negative” (Supplemental Fig. 1).

When we asked about the impact of COVID-19 on research opportunities and productivity of fellows in the 2019 to 2020 academic year, we received 178 responses. Of these, 13 (7.3%) responded “very positive,” 37 (20.8%) said “somewhat positive,” 74 (41.6%) said “neutral,” 40 (22.5%) said “somewhat negative,” and 14 (7.9%) said “very negative” (Supplemental Fig. 1).

When we asked about the impact of COVID-19 on the mentorship provided to fellows during the 2019 to 2020 academic year, we received 178 responses. Of these, 18 (10.1%) responded “very positive,” 32 (18%) said it was “somewhat positive,” 82 (46.1%) thought it was “neutral,” 41 (23%) considered the impact “somewhat negative,” and 5 (2.81%) reported it was “very negative” (Supplemental Fig. 1).

In response to the statement, “The COVID-19 pandemic has changed the experience for the current 2020-2021 fellowship class,” we received 171 responses. Of these 44 (25.7%) strongly agreed, 73 (42.7%) somewhat agreed, 29 (17%) were neutral, 18 (10.5%) somewhat disagreed, and 7 (4.1%) strongly disagreed (Supplemental Fig. 2).

In response to the statement “The effect of COVID-19 on fellowship training in the 2019 to 2020 academic year will negatively impact patient care provided by these fellows over the next 3-5 years in practice,” we received 171 responses. Of these, 4 (2.3%) strongly agreed, 24 (14%) somewhat agree, 28.07% (48/171) neutral, 29.82% (51/171) somewhat disagree, and 25.73% (44/171) strongly disagree (see Supplemental Fig. 3).

In response to the statement “The effect of COVID-19 on fellowship training in the 2019–2020 academic year will negatively impact patient care provided by these fellows beyond 5 years in practice,” we received 170 responses. Of these, 2 (1.2%) strongly agreed, 7 (4.1%) somewhat agreed, 28 (16.5%) were neutral, 51 (30%) somewhat disagreed, and 82 (48.2%) strongly disagreed (Supplemental Fig. 3).

In response to the statement “The effect of COVID-19 on fellowship training in the 2019–2020 academic year negatively impacted your graduate(s) ability to find employment,” we received 171 responses. Of these, 12 (7.6%) strongly agreed, 39 (22.8%) somewhat agreed, 35 (20.5%) were neutral, 34 (19.9%) somewhat disagreed, and 50 (29.2%) strongly disagreed (Supplemental Fig. 4).

In response to the statement, “COVID-19 negatively impacted the perceived value of fellowship training in a way that will decrease the desire for surgeons-in-training to pursue subspecialty fellowship training in the near future,” we received 171 responses. None of the participants strongly agreed with this statement. However, 11 (6.4%) somewhat agreed, 29 (17%) were neutral, 48 (28.1%) somewhat disagreed, and 83 (48.5%) strongly disagreed (Supplemental Fig. 5).

In response to the statement, “The COVID-19 pandemic will have long lasting (after the pandemic has resolved) effects on how orthopaedic surgery fellows are educated,” we received 171 responses. Of these 15 (8.8%) strongly agreed, 59 (34.7%) somewhat agreed, 37 (21.8%) were neutral, 30 (17.7%) somewhat disagreed, and 29 (17.1%) strongly disagreed (Supplemental Fig. 6).

In response to the statement, “During COVID-19 your fellowship program learned new ways to practice distanced education that you will continue to use even after COVID-related restrictions are lifted,” we received 171 responses. Of these, 68 (39.8%) strongly agreed, 79 (46.2%) somewhat agreed, 18 (10.5%) were neutral, 4 (2.3%) somewhat disagreed, and 2 (1.2%) strongly disagreed (Supplemental Fig. 7).

In response to the statement, “Fellows in the 2019–2020 academic year were better trained in virtual medicine as a result of COVID-19,” we received 171 responses. Of these, 46 (26.9%) strongly agreed, 71 (41.5%) somewhat agreed, 27 (15.8%) were neutral, 15 (8.8%) somewhat disagreed, and 12 (7.0%) strongly disagreed (Supplemental Fig. 8).

In response to the statement “Your program plans to make changes to the education of fellows going forward on a permanent basis as a result of the COVID-19 pandemic,” we received 171 responses. Of these, 23 (13.5%) strongly agreed, 72 (42.1%) somewhat agreed, 46 (26.9%) were neutral, 21 (12.3%) somewhat disagreed, and 9 (5.3%) strongly disagreed (Supplemental Fig. 9).

In response to the statement “You are considering terminating you fellowship program or decreasing the number of fellows per year because of your COVID-19 experience,” we received 171 responses. Of these, 2 (1.2%) strongly agreed, 5 (2.9%) somewhat agreed, 6 (3.5%) were neutral, 12 (7.0%) somewhat disagreed, and 146 (85.4%) strongly disagreed (Supplemental Fig. 10).

## Discussion

Our survey of orthopedic fellowship program directors found that while COVID-19 had a negative impact on fellowship training for the class of 2019 to 2020, as it did with most areas of life, a majority of directors believed it would not have a lasting negative effect on patient care and that some positive changes resulted, as well.

Our study was not without limitations. Our survey response rate was 33.7%, and we therefore lacked data from a majority of fellowship program directors. Also, because the survey was sent only to directors of accredited fellowship programs, it may not reflect the experiences of those who educate in nonaccredited programs or those who educate residents. Given that it was a one-time survey, the results allow only for a snapshot of a single point in time and cannot evolve as understanding evolves about the COVID-19 pandemic. Also, our survey was administered 8 months after the initial U.S. shutdowns due to COVID-19, and it may reflect attitudes that were significantly affected by the duration of the pandemic. In addition, although we attempted to survey program directors from across the nation, our results may have been tilted toward urban areas because training programs tend to follow population densities. Finally, because we had an extremely limited (4.44%) response from the directors of hand and wrist programs, our results might not be as applicable to those programs.

To more fully understand the effects of the COVID-19 pandemic, researchers may consider surveying additional orthopedic surgery educators such as faculty at nonaccredited fellowship programs or those who work in residency training programs. Additionally, after the pandemic ends, follow-up surveys at various time points could identify lasting changes to orthopedic surgeon education.

Positive changes observed in program directors’ responses included, for example, that only 16.4% of respondents believed the effects of COVID-19 on the 2019 to 2020 academic year would have a negative impact on patient care in the next 3 to 5 years. Even fewer (5.3%) said they thought there would be a negative impact on patient care beyond 5 years. Thus, a majority of our survey respondents believed that the main goal of fellowship training—patient care—would not be largely compromised. Another positive observation was that only 6.43% of respondents believed the perceived value of fellowship training had been negatively affected. Furthermore, only 4.1% of respondents reported considering decreasing or terminating their fellowship program, while 85.38% strongly disagreed with that statement.

In addition, 86% of program directors reported that, during the pandemic, their programs learned new ways to educate fellows. In fact, 68.3% of participants reported that fellows were better trained in virtual medicine, and 55.6% said their programs planned to make permanent changes to fellows’ education as a result of the pandemic. While medicine is often maligned for being slow to embrace new technology, these observations demonstrate educators’ embrace of new methods and recognition of their benefits. Lastly, only 30.4% of program directors agreed that COVID-19 negatively affected fellow’s ability to find employment. This is a reassuring percentage, but it was not clear whether fellows had fewer employment opportunities or were able to find employment in their desired geographic region or practice model. These results may therefore represent an attitude of “any employment is good employment.”

An interesting observation from our survey was the normal distribution of responses to questions regarding didactic and academic learning, research opportunities, and mentorship provided as a result of the COVID-19 pandemic. We may assume that many fellows, spending less time with patients and in the operating room, would look to participate in research opportunities, and similarly that training programs would find ways to improve didactic and academic learning; however, our survey did not confirm these assumptions. We speculate that the reported lack of increase in research and of training programs improving didactic and academic learning and mentorship may be due to fellows being repurposed to help provide care for COVID-19 patients or due to the fellows changing how they used their nonclinical time. These speculations require further investigation.

In conclusion, the COVID-19 pandemic resulted in many changes to orthopedic surgery fellowship training. Our survey found that program directors representing all subspecialties said that while the 2019 to 2020 academic year was negatively affected, they believed patient care provided by 2019 to 2020 fellows would not be negatively affected in the short- and medium-term. Program directors also reported positive outcomes such as learning new ways to educate fellows. Additionally, they believed that fellowship training maintains its educational value and only a very small minority planned to reduce training or positions.

## Supplemental Material

sj-pdf-1-hss-10.1177_15563316211012006 – Supplemental material for The Impact of COVID-19 on Orthopedic Surgery Fellowship Training: A Survey of Fellowship Program Directors Click here for additional data file. Supplemental material, sj-pdf-1-hss-10.1177_15563316211012006 for The Impact of COVID-19 on Orthopedic Surgery Fellowship Training: A Survey of Fellowship Program Directors by Braiden M. Heaps, Jeffrey R. Dugas and Orr Limpisvasti in HSS Journal®: The Musculoskeletal Journal of Hospital for Special Surgery

sj-pdf-2-hss-10.1177_15563316211012006 – Supplemental material for The Impact of COVID-19 on Orthopedic Surgery Fellowship Training: A Survey of Fellowship Program Directors Click here for additional data file. Supplemental material, sj-pdf-2-hss-10.1177_15563316211012006 for The Impact of COVID-19 on Orthopedic Surgery Fellowship Training: A Survey of Fellowship Program Directors by Braiden M. Heaps, Jeffrey R. Dugas and Orr Limpisvasti in HSS Journal®: The Musculoskeletal Journal of Hospital for Special Surgery

sj-zip-1-hss-10.1177_15563316211012006 – Supplemental material for The Impact of COVID-19 on Orthopedic Surgery Fellowship Training: A Survey of Fellowship Program Directors Click here for additional data file. Supplemental material, sj-zip-1-hss-10.1177_15563316211012006 for The Impact of COVID-19 on Orthopedic Surgery Fellowship Training: A Survey of Fellowship Program Directors by Braiden M. Heaps, Jeffrey R. Dugas and Orr Limpisvasti in HSS Journal®: The Musculoskeletal Journal of Hospital for Special Surgery
