# Supplementary material for: The Impact of COVID-19 on Orthopedic Surgery Fellowship Training: A Survey of Fellowship Program Directors
Source: HSS J. 2021 May 10;18(1):105–9. doi: 10.1177/15563316211012006 (PMC8753546; doi:10.1177/15563316211012006)
Supplement: sj-pdf-1-hss-10.1177_15563316211012006 – Supplemental material for The Impact of COVID-19 on Orthopedic Surgery Fellowship Training: A Survey of Fellowship Program Directors [file sj-pdf-1-hss-10.1177_15563316211012006.pdf]

Supplemental Figures for: Heaps et al. The Impact of COVID-19 on Orthopedic Surgery Fellowship Training: A Survey of Fellowship Program Directors. *HSS Journal.* 2021.

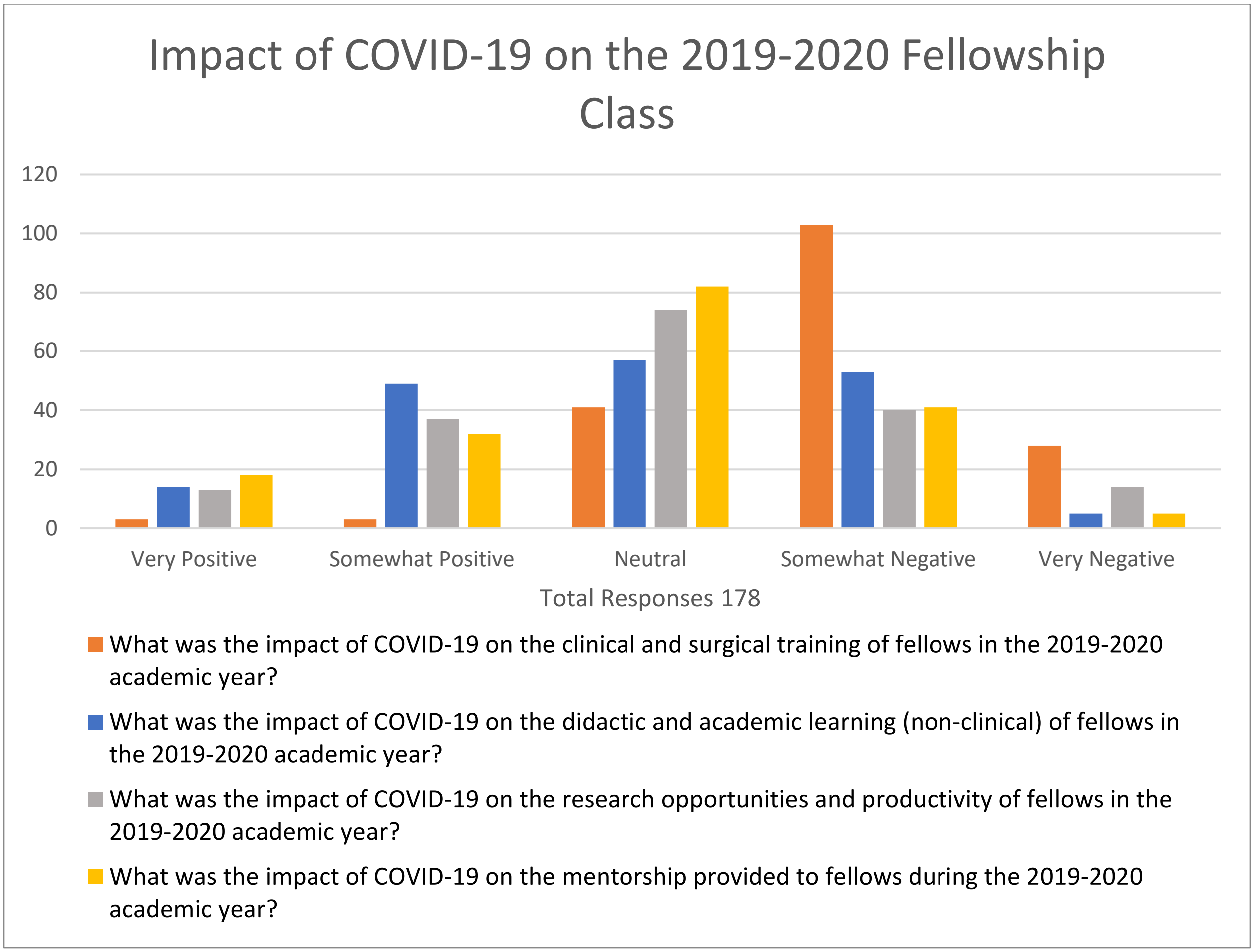

Supplemental Fig. 1. Responses to the questions: "What was the impact of COVID-19 on the clinical and surgical training of fellows in the 2019–2020 academic year?" "What was the impact of COVID-19 on the didactic and academic learning (non-clinical) of fellows in the 2019–2020 academic year?" "What was the impact of COVID-19 on the research opportunities and productivity of fellows in the 2019–2020 academic year?" "What was the impact of COVID-19 on the mentorship provided to fellows during the 2019–2020 academic year?"

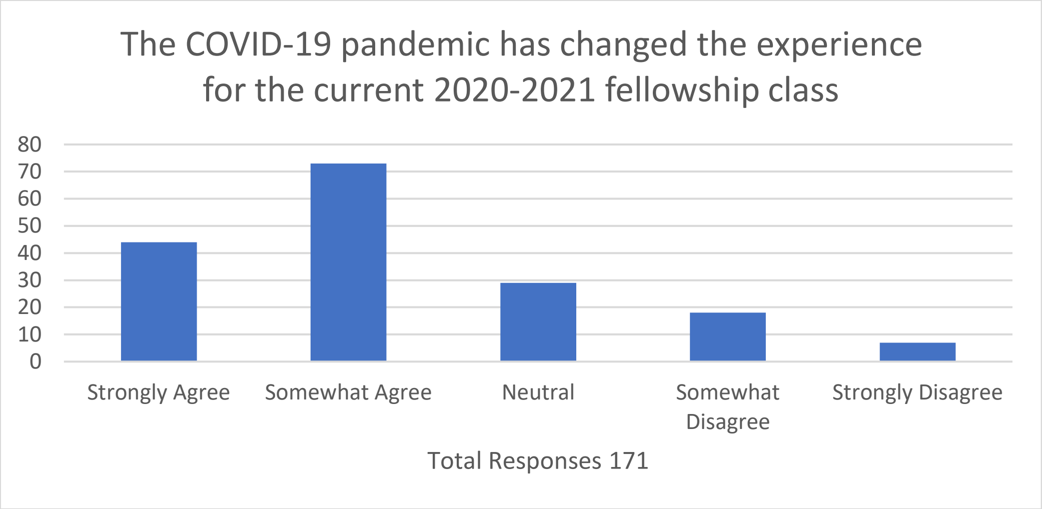

Supplemental Fig. 2. Responses to the statement: “The COVID-19 pandemic has changed the experience for the current 2020–2021 fellowship class.”

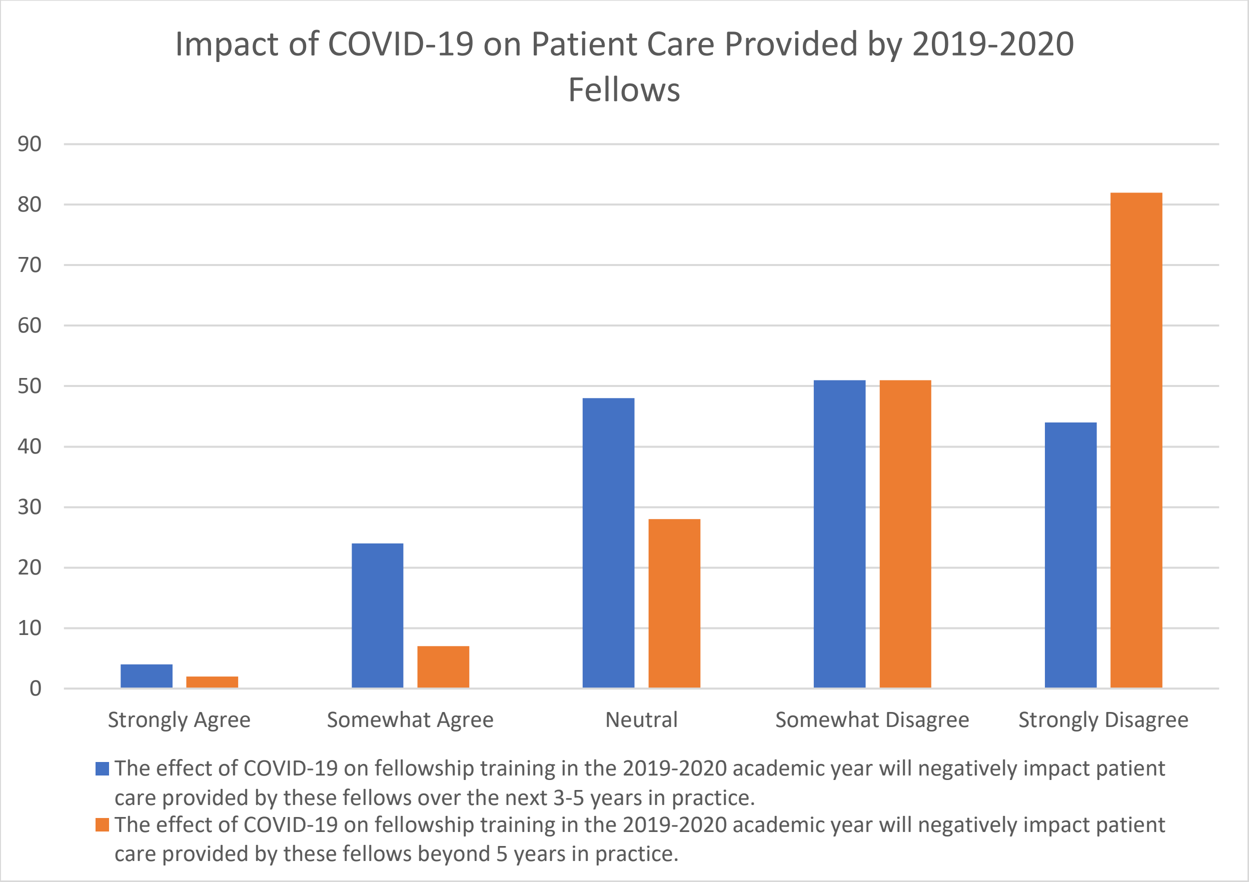

Supplemental Fig. 3. Responses to the statements: “The effect of COVID-19 on fellowship training in the 2019–2020 academic year will negatively impact patient care provided by these fellows over the next 3-5 years in practice.” “The effect of COVID-19 on fellowship training in the 2019–2020 academic year will negatively impact patient care provided by these fellows beyond 5 years in practice.”

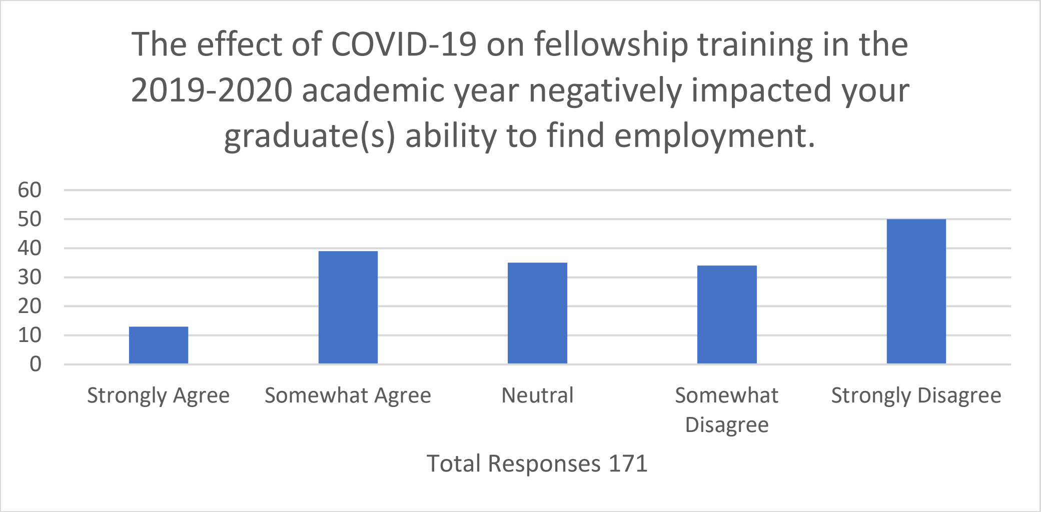

Supplemental Fig. 4. Responses to the statement: “The effect of COVID-19 on fellowship training in the 2019–2020 academic year negatively impacted your graduate(s) ability to find employment.”

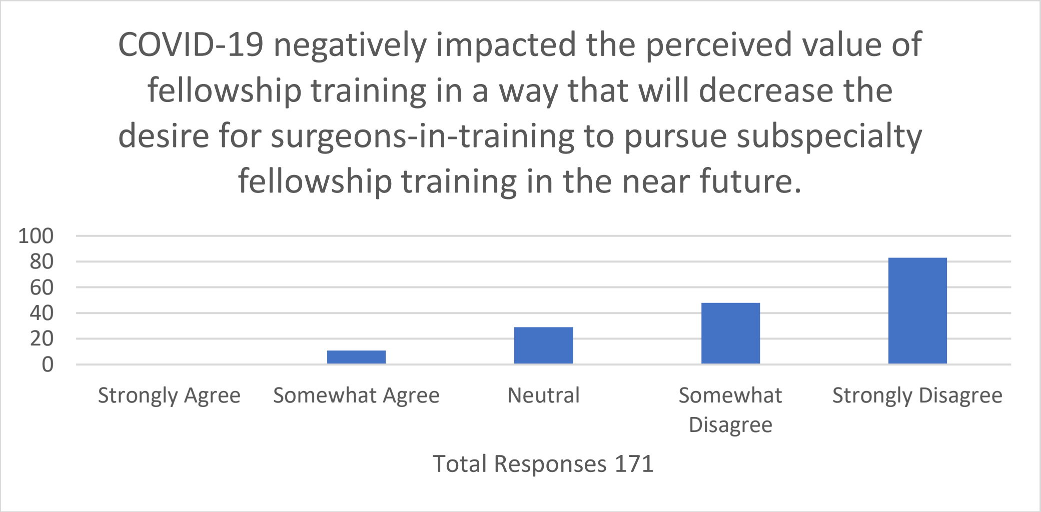

Supplemental Fig. 5. Responses to the statement: “COVID-19 negatively impacted the perceived value of fellowship training in a way that will decrease the desire for surgeons-in-training to pursue subspecialty fellowship training in the near future.”

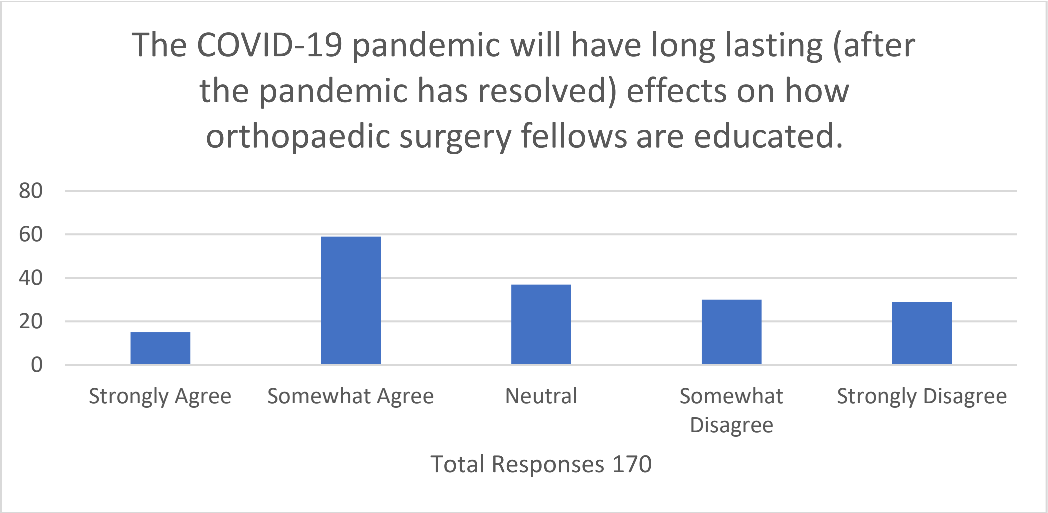

Supplemental Fig. 6. Responses to the statement: “The COVID-19 pandemic will have long lasting (after the pandemic has resolved) effects on how orthopaedic surgery fellows are educated.”

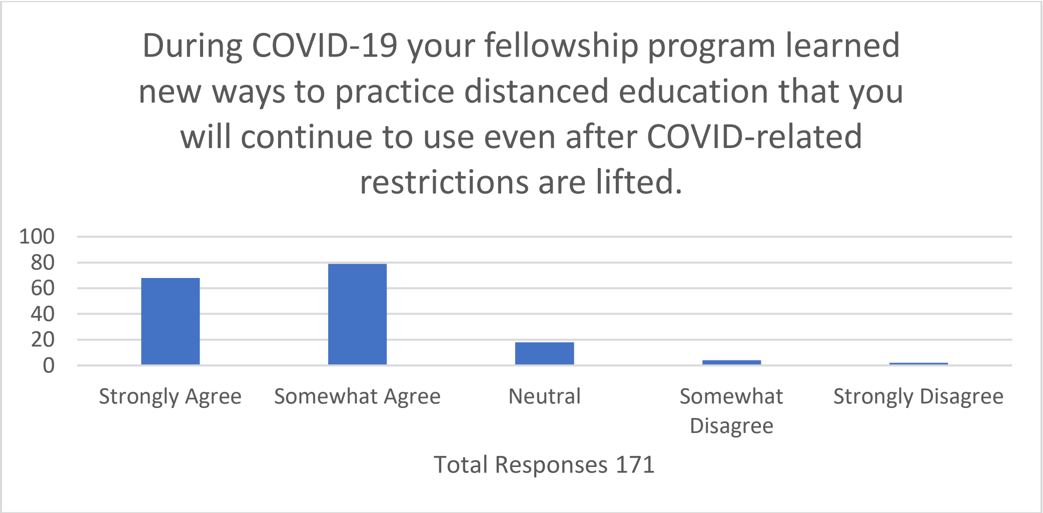

Supplemental Fig. 7. Responses to the statement: “During COVID-19 your fellowship program learned new ways to practice distanced education that you will continue to use even after COVID-related restrictions are lifted.”

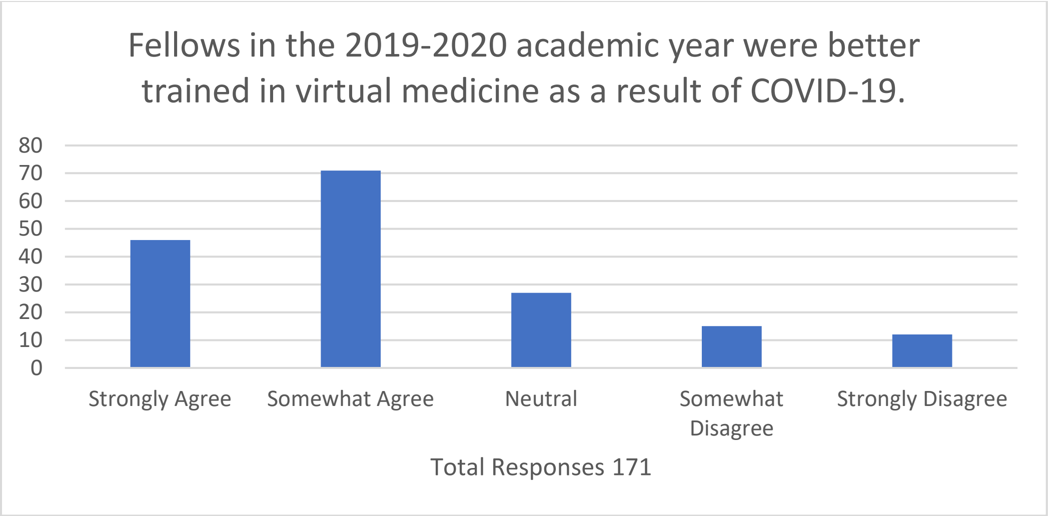

Supplemental Fig. 8. Responses to the statement: “Fellows in the 2019–2020 academic year were better trained in virtual medicine as a result of COVID-19.”

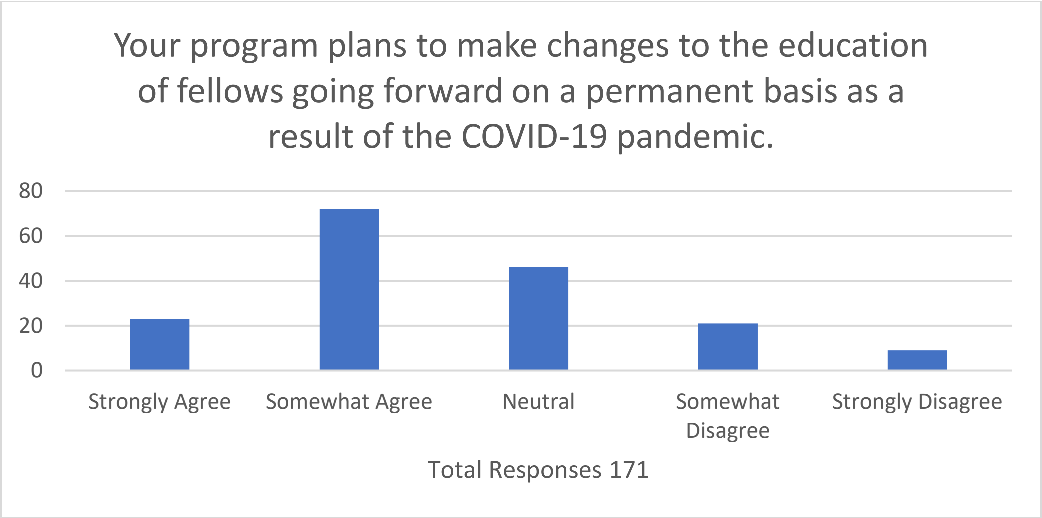

Supplemental Fig. 9. Responses to the statement: “Your program plans to make changes to the education of fellows going forward on a permanent basis as a result of the COVID-19 pandemic.”

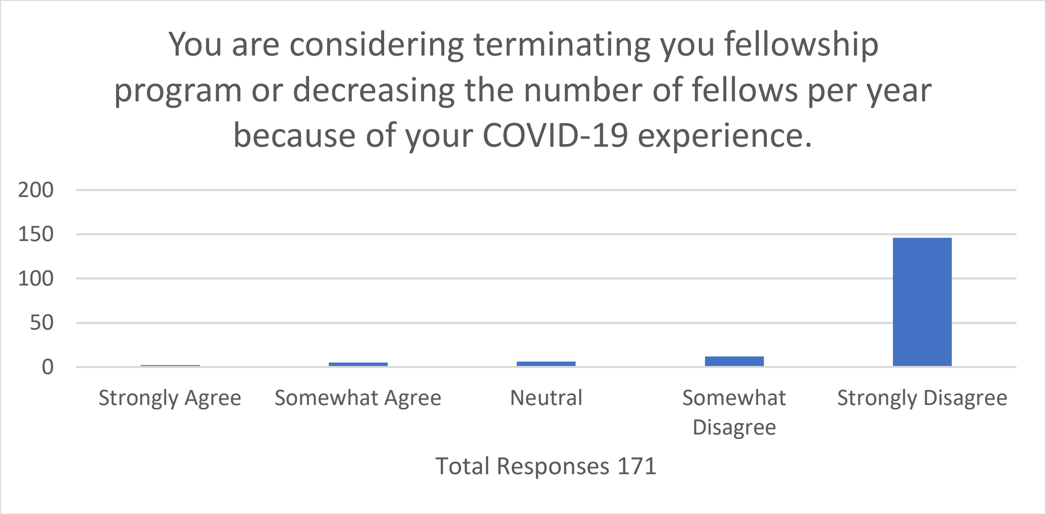

Supplemental Fig. 10. Responses to the statement: “You are considering terminating you fellowship program or decreasing the number of fellows per year because of your COVID-19 experience.”
