# Supplementary material for: The Impact of COVID-19 on Orthopedic Surgery Fellowship Training: A Survey of Fellowship Program Directors
Source: HSS J. 2021 May 10;18(1):105–9. doi: 10.1177/15563316211012006 (PMC8753546; doi:10.1177/15563316211012006)
Supplement: sj-pdf-2-hss-10.1177_15563316211012006 – Supplemental material for The Impact of COVID-19 on Orthopedic Surgery Fellowship Training: A Survey of Fellowship Program Directors [file sj-pdf-2-hss-10.1177_15563316211012006.pdf]

Supplemental Tables for: Heaps et al. The Impact of COVID-19 on Orthopedic Surgery Fellowship Training: A Survey of Fellowship Program Directors. *HSS Journal.* 2021.

Table 1. 18 item survey

1	Which subspecialty of orthopaedics is your fellowship program?
	Arthroplasty
	Foot and Ankle
	Hand and Wrist
	Hip Preservation
	Oncology
	Pediatrics
	Shoulder and Elbow
	Spine
	Sports Medicine
	Trauma
	Other (please specify)
2	How many fellows does your program train each year?
	1
	2
	3
	4
	5
	6
	7
	8
	9
	10+
3	In what region of the United States is your fellowship program located?
	Northeast
	Mid Atlantic
	Southeast
	Great Lakes Region
	Midwest
	Moutain West
	Southwest
	West Coast
4	In what type of practice model is your fellowship training program based?

Large Academic Center

Academic Center Affiliation

Private Practice

Community Based Practice

"Privademic"

Other (please specify)

5 What was the impact of COVID-19 on the clinical and surgical training of fellows in the 2019-2020 academic year?

Very Positive

Somewhat Positive

Neutral

Somewhat Negative

Very Negative

6 What was the impact of COVID-19 on the didactic and academic learning (non-clinical) of fellows in the 2019-2020 academic year?

Very Positive

Somewhat Positive

Neutral

Somewhat Negative

Very Negative

7 What was the impact of COVID-19 on the research opportunities and productivity of fellows in the 2019-2020 academic year?

Very Positive

Somewhat Positive

Neutral

Somewhat Negative

Very Negative

8 What was the impact of COVID-19 on the mentorship provided to fellows during the 2019-2020 academic year?

Very Positive

Somewhat Positive

Neutral

Somewhat Negative

Very Negative

9 The COVID-19 pandemic has changed the experience for the current 2020-2021 fellowship class.

Strongly Agree

Somewhat Agree

Neutral

Somewhat Disagree

Strongly Disagree

10 The effect of COVID-19 on fellowship training in the 2019-2020 academic year will negatively impact patient care provided by these fellows over the next 3-5 years in practice.

Strongly Agree

Somewhat Agree

Neutral

Somewhat Disagree

Strongly Disagree

11 The effect of COVID-19 on fellowship training in the 2019-2020 academic year will negatively impact patient care provided by these fellows beyond 5 years in practice.

Strongly Agree

Somewhat Agree

Neutral

Somewhat Disagree

Strongly Disagree

12 The effect of COVID-19 on fellowship training in the 2019-2020 academic year negatively impacted your graduate(s) ability to find employment.

Strongly Agree

Somewhat Agree

Neutral

Somewhat Disagree

Strongly Disagree

13 COVID-19 negatively impacted the perceived value of fellowship training in a way that will decrease the desire for surgeons-in-training to pursue subspecialty fellowship training in the near future.

Strongly Agree

Somewhat Agree

Neutral

Somewhat Disagree

Strongly Disagree

14 The COVID-19 pandemic will have long lasting (after the pandemic has resolved) effects on how orthopaedic surgery fellows are educated.

Strongly Agree

Somewhat Agree

Neutral

Somewhat Disagree

Strongly Disagree

15 During COVID-19 your fellowship program learned new ways to practice distanced education that you will continue to use even after COVID-related restrictions are lifted.

Strongly Agree

Somewhat Agree

Neutral

Somewhat Disagree

Strongly Disagree

16 Fellows in the 2019-2020 academic year were better trained in virtual medicine as a result of COVID-19.

Strongly Agree

Somewhat Agree

Neutral

Somewhat Disagree

Strongly Disagree

17 Your program plans to make changes to the education of fellows going forward on a permanent basis as a result o the COVID-19 pandemic.

Strongly Agree

Somewhat Agree

Neutral

Somewhat Disagree

Strongly Disagree

18 You are considering terminating you fellowship program or decreasing the number of fellows per year because of your COVID-19 experience.

Strongly Agree

Somewhat Agree

Neutral

Somewhat Disagree

Strongly Disagree

Table 2. Subspecialties of respondents

Subspecialty	Number of Responses	Percent of Responses	Number of Programs	Percent of Possible Programs
Arthroplasty	26	13.76	103	25.24
Foot and Ankle	31	16.40	50	62.00
Hand and Wrist	4	2.12	90	4.44
Hip Preservation	1	0.53	0	***
Oncology	5	2.65	20	25.00
Pediatrics	28	14.81	46	60.87
Shoulder and Elbow	17	8.99	31	54.84
Spine	20	10.58	74	27.03
Sports Medicine	31	16.40	88	35.23
Trauma	26	13.76	62	41.94
Other (please specify)	0	0.00	0	0.00
Total	189			
*** One program responded they are a hip preservation program; however, no programs have been accredited as hip preservation fellowships				

Table 3. Fellowship program demographics

	Number of Responses	Percentage of Responses
Number of fellows trained each year		
1	95	50.00
2	50	26.32
3	13	6.84
4	18	9.47
5	7	3.68
6	4	2.11
7	2	1.05
8	0	0.00
9	1	0.53
10+	0	0.00
Total	190	
In what region of the United States is your fellowship program located		
Northeast	47	24.74
Mid Atlantic	8	4.21
Southeast	38	20.00
Great Lakes Region	3	1.58
Midwest	38	20.00
Moutain West	11	5.79
Southwest	15	7.89
West Coast	30	15.79
Total	190	
In what type of practice model is your fellowship training program based		
Large Academic Center	118	62.43
Academic Center Affiliation	20	10.58
Private Practice	15	7.94
Community Based Practice	0	0.00
"Privademic"	31	16.40
Other (please specify)	5	2.65
Total	189	

Other Responses:	Academic Private Practice
	Shriners
	Hospital Employed Practice
	Standalone community based with Academic affiliation
	https://www.wellspan.org/news/story/wellspan-york-hospital-on-list-of-nations-best-teaching-hospitals/N6080
